# Seropositivity for Avian Influenza H6 Virus among Humans, China

**DOI:** 10.3201/eid2107.150135

**Published:** 2015-07

**Authors:** Li Xin, Tian Bai, Jian Fang Zhou, Yong Kun Chen, Xiao Dan Li, Wen Fei Zhu, Yan Li, Jing Tang, Tao Chen, Kun Qin, Jing Hong Shi, Rong Bao Gao, Da Yan Wang, Ji Ming Chen, Yue Long Shu

**Affiliations:** National Institute for Viral Disease Control and Prevention, Beijing, China (L. Xin, T. Bai, J.F. Zhou, Y.K. Chen, X.D. Li, W.F. Zhu, Y. Li, J. Tang, T. Chen, K. Qin, J.H. Shi, R.B. Gao, D.Y. Wang, Y.L. Shu);; China Animal Health and Epidemiology Center, Qingdao, China (J.M. Chen)

**Keywords:** avian influenza, H6N2, viruses, occupationally exposed populations, human infection, agricultural workers' diseases, poultry, China, seropositivity

**To the Editor:** Influenza virus subtype H6 was first isolated from a turkey in 1965 in the United States (*1*) and was subsequently found in other parts of the world (*2*). Over the past several decades, the prevalence of H6 virus has dramatically increased in wild and domestic birds (*2*–*4*). In China, highly pathogenic influenza A(H5N1), low pathogenicity influenza (H9N2), and H6 are the most prevalent avian influenza viruses among poultry (*5*). Although only 1 case of H6 virus infection in a human has been reported worldwide (*6*), several biological characteristics of H6 viruses indicate that they are highly infectious to mammals. Approximately 34% of H6 viruses circulating in China have enhanced affinity to human-like receptors (ɑ-2,6 NeuAcGal) (*2*). H6 viruses can also infect mice without prior adaptation (*2*,*7*), and some H6 viruses can be transmitted efficiently among guinea pigs (*2*). To evaluate the potential threat of H6 viruses to human health, we conducted a systematic serologic study in populations occupationally exposed to H6 viruses.

During 2009–2011, a total of 15,689 serum samples were collected from live poultry market workers, backyard poultry farmers, large-scale poultry farmers, poultry-slaughter factory workers, and wild bird habitat workers in 22 provinces in mainland China. A/chicken/Y94/Guangdong/2011 (H6N2), a representative isolate of predominant H6 viruses in mainland China, was used for the serologic testing (Technical Appendix Table 1). Hemagglutination inhibition (HI) assay was performed for all serum samples, and samples with an HI titer ≥20 were verified by a microneutralization (MN) assay, as indicated by World Health Organization guidelines (*8*). An MN result of ≥20 was considered positive.

The HI result was ≥20 for H6N2 virus in 298 of the 15,689 specimens, and the MN result was positive in 63 of the 298 specimens (overall seropositivity range 20–320, mean 32.7, 0.4%) (Technical Appendix Table 2). The proportion of group members who were seropositive differed significantly according to occupational exposure (p = 0.0125). Seropositivity was highest among workers in live poultry markets, backyard poultry farmers, and workers in wild bird habitats (0.66%, 0.42%, and 0.51%, respectively) (Table). According to χ^2 ^test results, seropositivity among workers in live poultry markets was significantly higher than that among large-scale poultry farmers (p = 0.0015, adjusted ɑ = 0.005. Analysis by unconditional logistic regression model showed that exposure to live poultry markets was a risk factor for human infection with avian influenza H6 virus (odds ratio 2.1, 95% CI 1.27–3.47).

**Table:** Seropositivity of occupationally exposed populations for the influenza (H6N2) virus, China, 2009–2011*

Population	Total no. serum samples	Mean titer for MN ≥20	No. serum samples with MN ≥20	Seropositivity (95% CI)	Odds ratio† (95% CI)
Total	15,689	32.70	63	0.40 (0.40–0.41)	
Occupation					
** Live poultry market**	3,950	43.08	26	0.66 (0.64–0.68)	2.10 (1.27–3.47)
** Poultry farm**	3,762	25.71	7	0.19 (0.18–0.19)	0.40 (0.18–0.87)
** Backyard poultry farm**	4,324	26.67	18	0.42 (0.40–0.43)	1.05 (0.61–1.82)
** Poultry slaughter factory**	1,235	30.00	2	0.16 (0.15–0.17)	0.38 (0.09–1.57)
** Wild bird habitat**	788	20.00	4	0.51 (0.47–0.54)	1.28 (0.47–3.54)
** Other**	1,630	23.33	6	0.37 (0.35–0.39)	0.91 (0.39–2.11)
Sex					
** F**	7,620	24.29	28	0.37 (0.36–0.38)	Reference
** M**	8,069	39.39	35	0.43 (0.42–0.44)	1.18 (0.72–1.94)
Age group, y					
** Children**, <14	74	–	0	0	0 (0)
** Youth**, 15–24	1,168	20.00	3	0.26 (0.24–0.27)	0.75 (0.19–3.00)
** Adult**, 25–59	1,2450	34.07	54	0.43 (0.43–0.44)	1.27 (0.54–2.94)
** Elderly**, >60	1,748	13.33	6	0.34 (0.33–0.36)	Reference
** No age record**	249	–	0	0	–
Geographic distribution					
** South**	10,522	32.00	50	0.48 (0.47–0.48)	Reference
** North**	5,167	35.38	13	0.25 (0.24–0.26)	0.59 (0.30–1.15)

*MN, microneutralization; **–, not applicable** †Odds ratios were calculated by using unconditional logistic regression model (SPSS 17.0, Armonk, NY, USA).

Seropositivity did not differ significantly among male and female persons tested (p = 0.08) (Table). No children were positive for the H6N2 virus. For other age groups, seropositivity ranged from 0.25% to 0.45%, but differences were not significant (p>0.05) (Table).

Of the 22 provinces from which serum specimens were collected, 11 were northern provinces and 11 were southern provinces. Positive specimens were detected in all southern provinces. In northern China, no seropositive results were detected in Henan, Liaoning, or Jilin Provinces. According to χ^2^ test results, seropositivity in southern China was significantly higher than seropositivity in northern China (p = 0.0375) (Table).

Human infection with influenza H6 virus in mainland China has not been reported, but 63 serum specimens tested in our study were positive for the H6 virus. This level of seropositivity is much higher than that for highly pathogenic avian influenza A(H5N1) virus, for which only 2 of the serum specimens we tested were positive (data not shown), but much lower than the seropositivity level for low pathogenicity avian influenza A(H9N2) virus; 3.4% of the samples tested were positive for A/Chicken/Hong Kong/G9/1997(H9N2)–like virus (data not shown). A previous US study has reported H6N2-positive antibodies in veterinarians (*9*). Our results and the veterinarian study indicate that the H6N2 virus could infect humans. 

In our study, positive samples were detected in 19 of 22 provinces and in all tested worker populations, suggesting that the H6 virus has been broadly circulating in birds in China. Live poultry market exposure is the major risk factor for human infection with avian influenza H6 virus. The limitation of this study is that antigen selection may not accurately detect neutralization antibodies for different subtypes of H6 viruses. Surveillance of the H6 virus in birds and occupationally exposed populations should be strengthened for pandemic preparedness.

**Technical Appendix.** Additional information regarding antigen selection and neutralization antibody titer of populations occupationally exposed to the avian influenza (H6N2) virus.
