# Supplementary material for: Seropositivity for Avian Influenza H6 Virus among Humans, China
Source: Emerg Infect Dis. 2015 Jul;21(7):1267–9. doi: 10.3201/eid2107.150135 (PMC4480397; doi:10.3201/eid2107.150135)
Supplement: Supplementary file 1 — Technical Appendix. Additional information regarding antigen selection and neutralization antibody titer of populations occupationally exposed to the avian influenza (H6N2) virus. [file 15-0135-Techapp-s1.pdf]

Article DOI: http://dx.doi.org/10.3201/eid2107.150135

Seropositivity for Avian Influenza H6 Virus among Humans, China

Technical Appendix

Technical Appendix Table 1. Levels of neutralization antibodies from serum specimens of occupationally exposed populations for the avian influenza (H6N2) virus, China*

Characteristic	Serum specimens	Serum specimens, MN ≥20 no. (%)	Serum specimens, MN ≥40 no. (%)	Serum specimens, MN ≥80 no. (%)	Serum specimens, MN ≥160 no. (%)	Serum specimens, MN = 320 no. (%)
Total population	15,689	63 (0.40)	14 (0.09)	5 (0.03)	2 (0.01)	1 (0.01)
Occupation						
Live poultry market worker	3,950	26 (0.66)	6 (0.15)	4 (0.10)	2 (0.05)	1 (0.03)
Poultry farmer	3,762	7 (0.19)	2 (0.05)	0 (0)	0 (0)	0 (0)
Backyard poultry farmer	4,324	18 (0.42)	4 (0.09)	1 (0.02)	0 (0)	0 (0)
Poultry-slaughter factory worker	1,235	2 (0.16)	1 (0.08)	0 (0)	0 (0)	0 (0)
Wild bird habitat worker	788	4 (0.51)	0 (0)	0 (0)	0 (0)	0 (0)
Others	1,630	6 (0.37)	1 (0.06)	0 (0)	0 (0)	0 (0)
Gender						
Female	7,620	28 (0.37)	4 (0.05)	1 (0.01)	0 (0)	0 (0)
Male	8,069	35 (0.43)	10 (0.12)	4 (0.05)	2 (0.02)	1 (0.01)
Age groups						
Children (≤14)	74	0 (0)	0 (0)	0 (0)	0 (0)	0 (0)
Youth (15–24)	1,168	3 (0.26)	0 (0)	0 (0)	0 (0)	0 (0)
Adult (25–59)	12,450	54 (0.43)	12 (0.10)	5 (0.04)	2 (0.02)	1 (0.01)
Elderly (≥60)	1,748	6 (0.34)	2 (0.11)	0 (0)	0 (0)	0 (0)

Characteristic	Serum specimens	Serum specimens, MN ≥20 no. (%)	Serum specimens, MN ≥40 no. (%)	Serum specimens, MN ≥80 no. (%)	Serum specimens, MN ≥160 no. (%)	Serum specimens, MN = 320 no. (%)
No age record	249	0 (0)	0 (0)	0 (0)	0 (0)	0 (0)
Geographic distribution						
South	10,522	50 (0.48)	10 (0.10)	4 (0.04)	1 (0.01)	1 (0.01)
North	5,167	13 (0.25)	4 (0.08)	1 (0.02)	1 (0.02)	0 (0)

*MN, microneutralization; specimens were tested by using MN assay.

Technical Appendix Table 2. Antigenic analysis of randomly selected H6N2 viruses and an H9N2 virus circulating in China*

Virus Strain	Ferret antisera		
	A/chicken/Y94/Guangdong/2011	A/chicken/YF6/Guangdong/2011	A/chicken/AK4/Anhui/2011
A/chicken/Y94/Guangdong/2011(H6N2)	**2,560**	2,560	<10
A/chicken/ YF6/Guangdong/2011(H6N2)	2560	**5,120**	<10
A/chicken/AK4/Anhui/2011(H9N2)	<10	<10	**10,240**

*Antigenic analysis was performed with hemagglutination assay by using 1% turkey red blood cells. Two representative avian influenza (H6N2) viruses located in the major clade and 1 avian influenza (H9N2) virus were randomly selected. Ferret antisera raised against these 3 viruses were used. No cross reaction occurred between H9N2 and H6N2 viruses. The 2 H6N2 viruses were antigenically similar. Homologous titers are in bold.

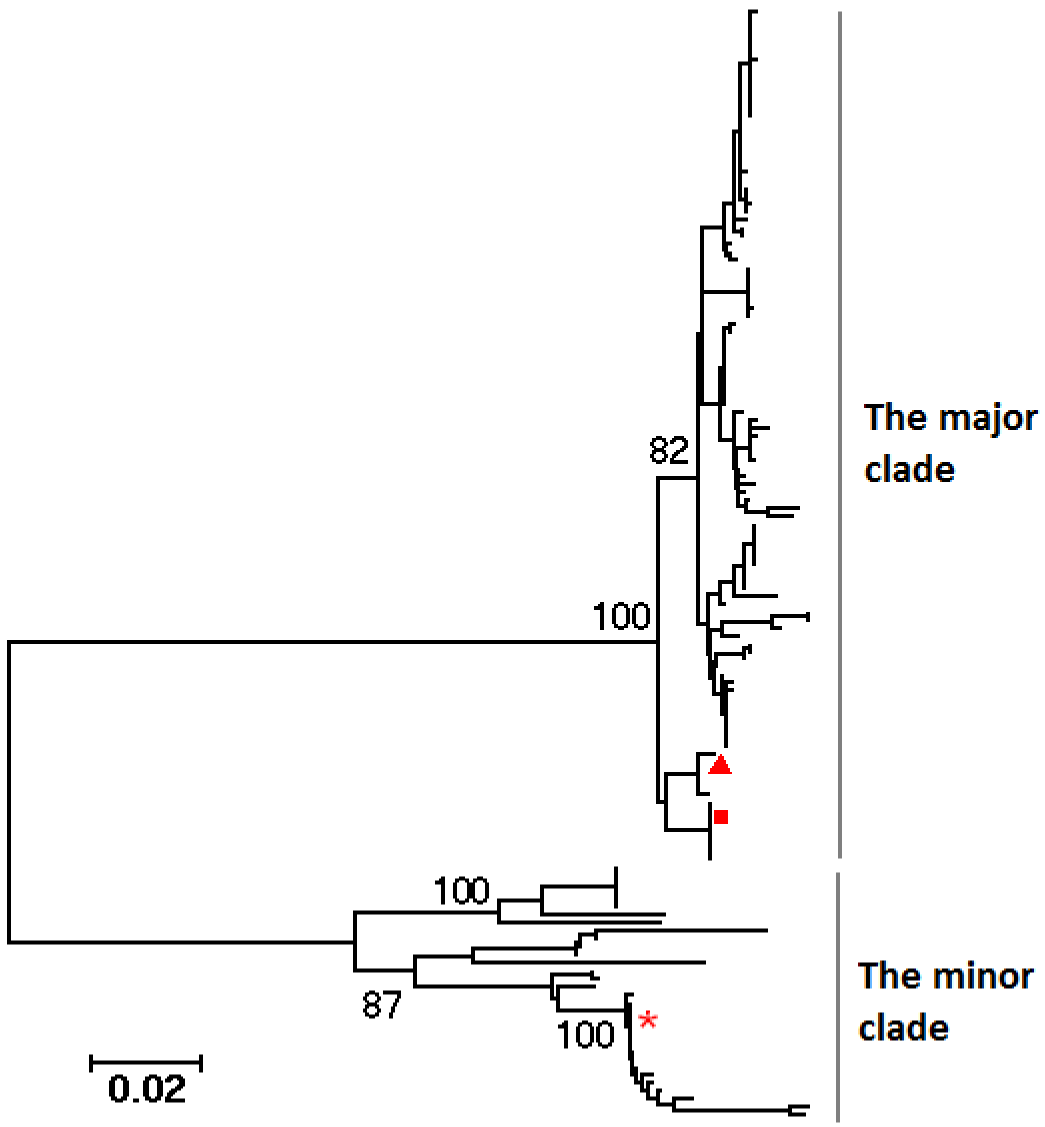

Technical Appendix Figure 1. Phylogenetic tree of H6 avian influenza viruses circulating in poultry in China in 2011 on the basis of HA1 domain sequences. Of 142 H6 subtype viruses isolated from birds in China in 2011, 140 were sequenced and classified into 2 clades. ▲represents A/chicken/Guangdong/Y94/2011(H6N2), ■ represents A/chicken/Guangdong/YF6/2011(H6N2), and * represents A/environment/Hunan-changsha/14/2011. Marked viruses were randomly selected for antigenic analysis. The phylogenetic tree was generated by the neighbor-joining method using Mega 6.0 (http://www.megasoftware.net). The bootstrap values of the main branch are shown. The scale bar indicates nucleotide substitutions per site.

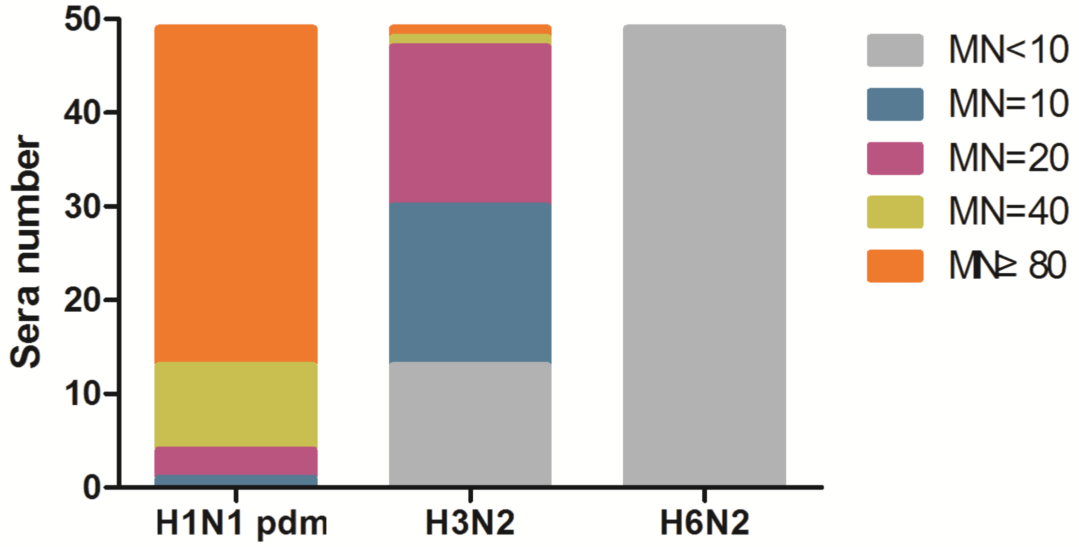

Technical Appendix Figure 2. Cross reaction of seasonal H1N1 pdm and H3N2 viruses with the H6N2 avian influenza virus by MN assay. Of sera samples positive for seasonal influenza H1N1 pdm and H3N2 viruses by HI assay, 49 were randomly selected for a cross-reaction analysis by using the MN assay. Results showed that sera testing positive for H1N1 pdm or H3N2 all had an MN titer <10 for H6N2, indicating no cross-reactions between H6N2 avian influenza and H1N1 pdm/H3N2 viruses. The H1N1pdm, H3N2, and H6N2 antigens used were A/California/07/2009(H1N1), A/Brisbane/10/2007(H3N2), and A/chicken/Y94/Guangdong/2011(H6N2), respectively. MN, microneutralization.
